# Buckling Analysis of CNT-Reinforced Polymer Composite Beam Using Experimental and Analytical Methods

**DOI:** 10.3390/ma16020614

**Published:** 2023-01-09

**Authors:** Emrah Madenci, Yasin Onuralp Özkılıç, Ceyhun Aksoylu, Muhammad Rizal Muhammad Asyraf, Agusril Syamsir, Abu Bakar Mohd Supian, Nicolay Mamaev

**Affiliations:** 1 Department of Civil Engineering, Necmettin Erbakan University, Konya 42090, Turkey; 2 Department of Civil Engineering, Konya Technical University, Konya 42090, Turkey; 3 Engineering Design Research Group (EDRG), Faculty of Mechanical Engineering, Universiti Teknologi Malaysia, Johor Bahru 81310, Johor, Malaysia; 4 Centre for Advanced Composite Materials (CACM), Universiti Teknologi Malaysia, Johor Bahru 81310, Johor, Malaysia; 5 Institute of Energy Infrastructure (IEI), Universiti Tenaga Nasional, Jalan IKRAM-UNITEN, Kajang 43000, Selangor, Malaysia; 6 World-Class Research Center “Advanced Digital Technologies”, State Marine Technical University, 190121 Saint-Petersburg, Russia

**Keywords:** carbon nanotube, polymer composites, buckling analysis, laminated composites

## Abstract

The aim of this article was to investigate the effect of carbon nanotubes (CNTs) on the buckling behavior of fiber-reinforced polymer (FRP) composites. The materials used included three layers: carbon-fiber-reinforced polymer (CFRP), epoxy and CNTs. A set of mechanical tests, such as compression and buckling tests, was performed, and also analytical solutions were developed. Damage analysis was also carried out by controlling the damage initiation and crack progression on the composite samples. Experimental results revealed that using 0.3% with CNT additives enhanced the buckling performance of the composite. Finally, the average load-carrying capacity for the clamped–clamped boundary condition was 268% higher in the CNT samples and 282% higher in the NEAT samples compared to the simple–simple condition.

## 1. Introduction

CNTs are utilized as important additive materials for high-performance structural composites, which have a lot of application potential [1]. The extreme interest in nanostructures among scientists and researchers is leading to the rapid development and characterization of nanocomposite material. When the dimensions of these structures are very small, at micro and nano scale, it has been shown using both experimental and atomistic simulation that the size effect on mechanical properties gains importance [2]. A single-walled carbon nanotube (SWCNT) is formed as a cylinder with a diameter of 1 nm. A multi-walled carbon nanotube (MWCNT) consists of a concentric form and a 0.35 nm separated array of cylinders from 2 to 100 nm in diameter and tens of microns in length [3]. Fidelus et al. [4] reported the experimental elastic properties of SWCNT and MWCNT. The elastic modulus of CNTs is reported to vary in a wide range from 200 GPa to 5.6 TPa [5].

Composite materials consist of matrix and reinforcement phases, and it is a material system that achieves distinctive features that no single component can achieve alone [6,7,8,9,10,11,12,13,14,15]. Generally, composites are formed by dispersing the reinforcement phase in a matrix phase. The reinforcement phase, which is a load-bearing element, determines the strength of the composite and its structural properties, such as thermal stability, electrical thermal conductivity, etc. It is effective in determining other functions such as the matrix phase holding the reinforcement materials together, transferring the load to the reinforcements and protecting the reinforcements from chemical and mechanical damage [15]. The matrix phases may be metals, polymers or ceramics, while the fibers may be particles or whiskers [16,17,18,19]. The performance of load-bearing fibers in composite constructions is significantly influenced by the matrix’s mechanical properties [20]. The matrix’s presence ensures that the load is evenly distributed among all fibers. The mechanical characteristics of the matrix and the bond strengths between the fiber and the matrix are significant parameters that define the strength of the composite structure in the direction perpendicular to the fiber orientations (i.e., toward the length of the fibers) [6,21,22,23]. The matrix is more ductile and less strong than fiber. The design of composite constructions should take this aspect into account. A break in either the fiber or the matrix could spread in all directions without changing direction if the shear strength of the matrix and the bond strength between the matrix and the fiber are sufficiently high [24]. In this case, since the composite acts as a brittle material, the rupture surface shows a clean and shiny structure. If the bond strength is too low, the fibers behave like a fiber bundle in the void, and the composite weakens. At a moderate bond strength, a crack in the transverse direction starting from the fiber or matrix may return to the fiber/matrix interface and progress in the fiber direction. In this case, the composite exhibits a fibrous surface, such as in the rupture of ductile materials.

Carbon-fiber-reinforced epoxy resin composites are polymeric composites that are used extensively in aerospace and military fields due to their durability and lightness. Carbon fiber shows good mechanical properties [25,26,27] as well as good fatigue resistance and heat resistance. When a polymer is used as the matrix phase, low shrinkage, good adhesion, excellent mechanical properties and chemical resistance are achieved. In addition to these advantages, carbon fiber also has some disadvantages. For example, composite laminates using carbon fiber exhibit relatively weak interlayer bond properties. This is due to the low fracture toughness of epoxy resins caused by the high crosslink density. To find a solution to this disadvantage, the excellent thermal, electrical, mechanical and functional properties of CNTs have been the subject of significant research in recent years. CNT-based fiber-reinforced polymer (FRP) composite materials have become the focus of attention in various applications [28]. Numerous analytical, experimental and numerical studies have been performed to demonstrate the ultra-high strength-to-weight ratio and stiffness-to-weight ratio of CNT-reinforced composite (CNTRC) structures [29,30,31,32,33,34,35,36,37,38]. Depending on the matrix class, various composite materials with CNT reinforcement have been produced. Recently, studies including bending, buckling and vibration analyses of polymer matrix composites reinforced with carbon fibers and CNTs have been of interest. Peigney et al. [39] and Zhan et al. [40] created several examples of CNTs-reinforced ceramic resin, and Milo et al. [41] embedded CNTs in a polymer matrix. In larger matrix layers, reinforcing by adding a little amount of nanotube leads to a significant gain in beam stiffness, and SWCNT buckles at reduced bending angles and greater flattening ratios according to published data on CNTRCs [42]. CNTs have been successfully incorporated into FRPs, providing increased strength and stiffness compared to standard carbon fibers. Shahbaz [43] presented a study on the effect of CNTs on hybrid GFRP/CFRP composites. He used two different techniques to add CNTs to hybrid glass/carbon-fiber-reinforced composites. Madenci [44] performed free vibration analysis of FG-CNT composite beams. He estimated the effective material properties of nanobeams using the mixing rule. Qian et al. [45] showed that, by adding different percentages of CNT (about 1% by weight) to the matrix material, in their research on the sample obtained, the hardness of the composite could be increased by between 36% and 42%, and the tensile strength could be increased by 25%. Zhu et al. [46] obtained the stress–strain curve of 1 and 4 wt% CNT-reinforced epoxy resin. The authors found a 30–70% increase in the elastic modulus for these weight fractions. Tarfaoui et al. [47] investigated the effect of CNT in CNTRCs with different volume fractions. They observed that an increase in the CNT volume fraction decreased (0.5–2%) the material properties after a certain value. Gouda et al. [48] investigated the impact of CNT on a hybrid composite GFRP/CFRP at various volume ratios.

The low mechanical properties of the matrix and the weakness of the CFRP interface are the main reasons for improving the interfacial behavior and epoxy properties of CFRP composites by the addition of nanoparticles. Doping large-scale composites with another material such as CNT can offset the disadvantage of single reinforcement. With the addition of CNTs to carbon-fiber composites, structural improvements are expected in through-the-thickness properties, particularly interlayer strength and toughness, which can lead to increased damage resistance and damage tolerance. On the other hand, the addition of CNTs may raise other issues that need to be considered; the dispersion of carbon nanotubes in the matrix phase and the coupling between the matrix and the CNTs should be examined.

A crucial element of structural and continuum mechanics, stability theory, has limitless applications in civil, mechanical, aerospace, naval and nuclear engineering [49]. The impact of CNTs added to conventional polymer-mix-based composites during buckling analysis is examined in this research. Low-CNT volume fractions can visibly change the mechanical behavior of composites. Experimental tensile and compression tests are used to obtain the mechanical properties of CNTRC beam, and theoretical and experimental buckling analyses are presented. Mechanical properties such as density and Young’s moduli are obtained through experimental tests; thus, the accurate predictions of such composite beams are significant research objectives. A buckling analysis is performed by applying Timoshenko theory. The governing equations of motion are derived from the theory using Hamilton’s principle. In addition to the experimental tests, calculations are made with the mixture rule model. Within the scope of the study, neat epoxy and 0.3% by weight CNTRC carbon-fiber composite samples were prepared.

## 2. Production of Materials

**Figure 1:**
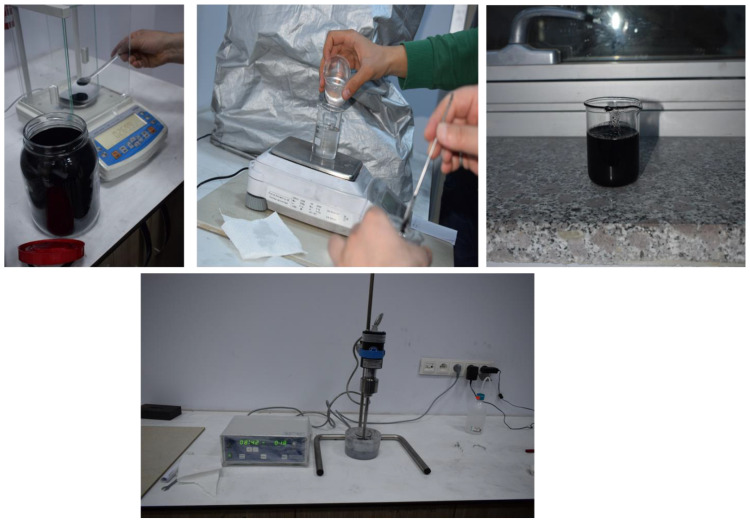
CNTs in epoxy resin and using the mixture of resin/CNT as the matrix of the composite.

**Figure 2:**
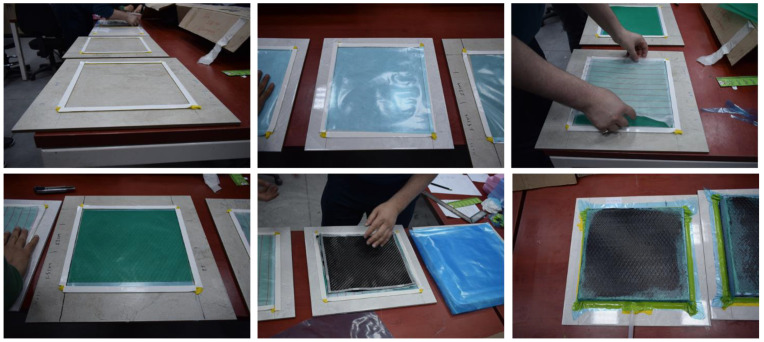
Manufacture of CNTRC composite material.

In order to manufacture composite material using the hand layup method, fabric layers were manually placed into the mold, and the epoxy resin was applied between each layer of the carbon fabric to completely coat the layer. By using a small roller, the air was trapped and evacuated. The process was performed at room temperature, and no curing process was carried out. By uniformly combining CNTs in epoxy resin and using the mixture of resin/CNT as the matrix of the composite, CNTRC was manufactured (Figure 1). The industrial method is a production method that uses atmospheric pressure to compress resin-impregnated layers together. The equipment that was used included a vacuum pump, peel ply, sealant tape, bagging films, carbon fabric, epoxy resin and infusion mesh (Figure 2). After layup was finished, the peel ply was applied over the layers to create a clean surface, and, over the peel ply, a layer of infusion mesh was placed to assist the flow of resin across and throughout the laminate during the resin infusion process. Layers were sealed in an airtight vacuum bag, and epoxy resin was transferred by force of vacuum and passed through the fabric layers. Like the previous method, the process was performed at room temperature, and no curing was carried out. Some of the advantages of this method are that no air is trapped, and uniform resin distribution is achieved.

**Figure 3:**
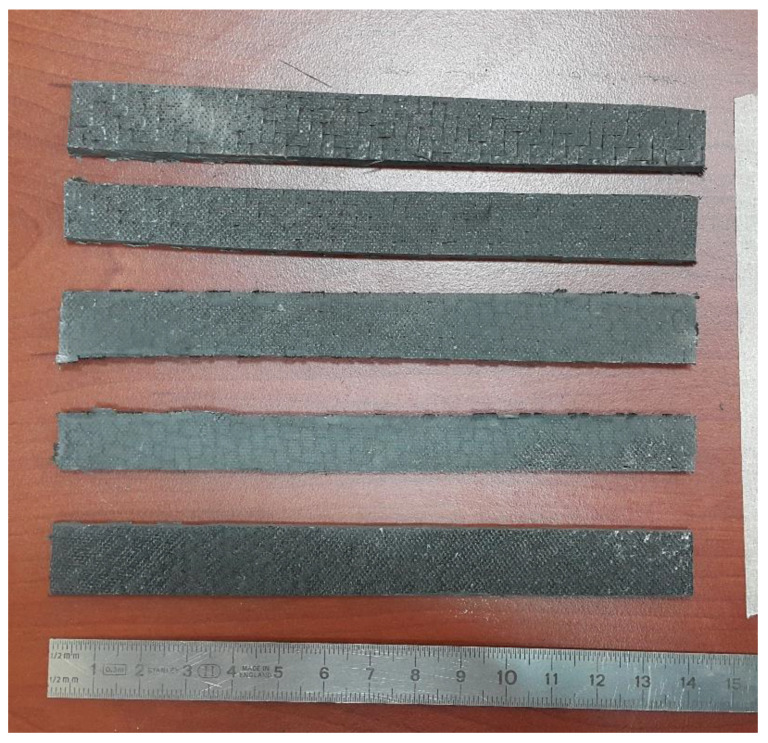
CNTRC composite samples.

The composite material was manufactured by three layers of carbon fibers as well as resin epoxy (Figure 3).

The epoxy resin utilized in the manufacturing had a viscosity of 600–900 mPas and was two phase, with 80–90% diglycidyl ether bisphenol A, and it contained a mixture of 10–20% aliphatic diglycidyl ether. Multi-walled carbon nanotubes (MWCNTs) were preferred in the current study due to their low cost and homogeneous dispersibility in epoxy resins compared to MWCNTs. The mechanical properties of the MWCNTs were a diameter of 5–50 nm and a length of 10–30 µm. In this study, 200 gr carbon-fiber fabric produced from Tenax-E HTA 40 3k yarn in plain weave type was used. Carbon-fiber fabric (CFRP) is ideal for applications where lightness, strength and carbon are important. The mechanical properties obtained from the manufacturer of these materials are shown in Table 1.

## 3. Analytical Methods

### 3.1. Effective Properties of CNTRC

Young’s modulus, Poisson’s ratio and shear modulus are a few examples of the material parameters that need to be understood in order to predict the mechanical behavior of CNTRCs under buckling stress. In this section, the theoretical mixture rule model utilized to determine the properties of composites (CNT + epoxy) is defined as [50]:

(1) $$E_{{}_{11}}^{epoxy+CNT}=\eta_{1}V_{CNT}E_{11}^{CNT}+V_{m}E_{m}$$

(2) $$\frac{\eta_{2}}{E_{22}}=\frac{V_{CNT}}{E_{22}^{CNT}}+\frac{V_{m}}{E_{22}^{m}}$$

(3) $$\frac{\eta_{3}}{G_{12}}=\frac{V_{CNT}}{G_{12}^{CNT}}+\frac{V_{m}}{G_{m}}$$

(4) $$\nu_{12}=V_{CNT}\nu_{12}^{CNT}+V_{m}\nu_{m}$$

(5) $$\rho =V_{CNT}\rho_{CNT}+V_{m}\rho_{m}$$

where $E_{11}^{CNT}$, $E_{22}^{CNT}$ and $G_{12}^{CNT}$ are Young’s and shear modulus of the single-walled *CNT*, respectively, and $E_{m}$ and $G_{m}$ represent the corresponding properties of the isotropic matrix. To account for the scale-dependent material properties, $\eta_{i}(i=1,2,3)$ is the *CNT*/matrix efficiency parameters which can be determined by matching the effective properties of the CNTRC observed from the molecular dynamics simulations with the predictions of the extended rule of mixture. $\rho_{CNT}$ and $\rho_{m}$ are the mass densities of the *CNT* and matrix, respectively. $V_{CNT}$ and $V_{m}$ are the volume fractions of the *CNT* and the matrix, respectively, and are related by:

(6) $$V_{CNT}+V_{m}=1$$

The mixture rule was developed for the mechanical properties of the fiber and matrix (*CNT* + *epoxy*) as follows [51]:

(7) $$E_{{}_{11}}^{composite}=V_{fiber}E_{11}^{fiber}+V_{m}E_{{}_{m}}^{\left(epoxy+CNT\right)}$$

(8) $$\nu_{{}_{12}}^{composite}=V_{fiber}\nu_{12}^{fiber}+V_{m}\nu_{{}_{m}}^{epoxy+CNT}$$

### 3.2. Governing Equations of CNTRC

The displacement field consisting of the axial displacement *u* and the transverse displacement *w* based on the Timoshenko beam theory of the forms are [52,53,54]:

(9) $$\begin{array}{l} u(x,z,t)=u_{0}(x,t)+z\phi_{x}(x,t)\\ v(x,z,t)=0\\ w(x,z,t)=w_{0}(x,t) \end{array}$$

where $u_{0}$ and $w_{0}$ are the axial and transverse displacements in the mid plane (*z* = 0) of the beam, respectively. $\phi_{x}$ is the mid-plane rotation of the transverse normal about the *y*-axis.

The strain–displacement relations can be evaluated as:

(10) $$\begin{array}{l} \varepsilon_{xx}=\frac{\partial u_{0}}{\partial x}+z\frac{\partial \phi_{x}}{\partial x}\\ \gamma_{xz}=\frac{\partial w_{0}}{\partial x}+\phi_{x} \end{array}$$

Hamilton’s principle can be written as [55]:

(11) $$\int_{t_{1}}^{t_{2}}\left(\delta U+\delta V-\delta K\right)dt=0$$

where *K* denotes the kinetic energy given by [56]:

(12) $$\begin{array}{ll} \delta K & =\int_{0}^{L}\int_{A}\rho (z)\left[u\delta u+w\delta w\right]dAdx\\ & =\int_{0}^{L}\left[I_{0}(u_{0}\delta u_{0}+w_{0}\delta w_{0})+I_{1}(\phi_{x}\delta u_{0}+u_{0}\delta \phi_{x})+I_{2}\phi_{x}\delta \phi_{x}\right]dx \end{array}$$

in which *I_i_*(i = 0, 1, 2) is the mass moment of inertia given by:

(13) $$I_{i}=\int_{A}\rho (z)z^{i}dA\;\;\;\;\;(i=0,1,2)$$

and δU is the virtual variation of the total strain energy:

(14) $$\begin{array}{ll} \delta U & =\int_{0}^{L}\int_{A}\left(Q_{11}\varepsilon_{xx}\delta \varepsilon_{xx}+Q_{55}\gamma_{xz}\delta \gamma_{xz}\right)dAdx\\ & =\int_{0}^{L}\left(N_{x}\frac{du_{0}}{dx}-M_{x}\frac{d\delta \phi_{x}}{dx}+Q_{x}(\frac{d\delta w_{0}}{dx}-\delta \phi_{x})\right)dx \end{array}$$

and δV is the virtual work performed by the transverse load *q* and axially compressive force $N_{x0}$, represented as:

(15) $$\delta V=-\int_{0}^{L}\left(q\delta w_{0}+N_{x0}\frac{\partial w_{0}}{\partial x}\frac{\partial \delta w_{0}}{\partial x}\right)dx$$

where the stress-related effects are as follows:

(16) $$\begin{array}{l} N_{x}=\int_{A}\sigma_{xx}dA\\ M_{x}=\int_{A}\sigma_{xx}zdA\\ Q_{z}=\int_{A}\sigma_{xz}dA \end{array}$$

The simplified stress–strain constitutive equations are given below:

(17) $$\begin{array}{l} \sigma_{xx}=Q_{11}\varepsilon_{xx}\\ \sigma_{xz}=Q_{55}\gamma_{xz} \end{array}$$

where

(18) $$\begin{array}{l} Q_{11}=\frac{E_{1}}{1-\vartheta_{12}\vartheta_{21}}\\ Q_{55}=G_{13} \end{array}$$

Substituting Equations (10) and (17) into Equation (16), the following is a list of all the stress resultants that may be expressed as material stiffness components and displacements:

(19) $$\begin{array}{l} \frac{\partial \phi_{x}}{\partial x}=D_{11}^{*}M_{x}\\ \frac{\partial w_{0}}{\partial x}+\phi_{x}=\frac{1}{k_{s}}A_{55}^{*}Q_{x} \end{array}$$

where ks=56 is the shear correction factor, and $D_{11}^{*}$ and $A_{55}^{*}$ are the elements of the inverse matrix of $D_{11}$ and $A_{55}$.

(20) D11=∫−h2h2Q11z2dz=∑k=1N∫zkzk+1Q11(k)z2dzA55=∫−h2h2Q55dz=∑k=1N∫zkzk+1Q55(k)dz

The following equations of motion are obtained for symmetrically laminated beams by using the integration-by-parts approach and gathering the coefficients of $\delta w_{0}$ and $\delta \varphi_{x}$ when the in-plane displacements $u_{0}$ are zero.

(21) $$\begin{array}{l} \frac{\partial Q_{x}}{\partial x}+{\hat{N}}_{x0}\frac{\partial^{2}w_{0}}{\partial x^{2}}+q=I_{0}\frac{\partial^{2}w_{0}}{\partial t^{2}}\\ \frac{\partial M_{x}}{\partial x}-Q_{x}=I_{{}_{2}}\frac{\partial^{2}\phi_{x}}{\partial t^{2}} \end{array}$$

The equation of motion, Equation (21), may be expressed using Equation (19) in terms of displacements as:

(22) $$\begin{array}{l} k_{s}G_{xz}A(\frac{\partial^{2}w_{0}}{\partial x^{2}}+\frac{\partial \phi_{x}}{\partial x})+b{\hat{N}}_{x0}\frac{\partial^{2}w_{0}}{\partial x^{2}}+\hat{q}={\hat{I}}_{0}\frac{\partial^{2}w_{0}}{\partial t^{2}}\\ E_{xx}I_{yy}\frac{\partial^{2}\phi_{x}}{\partial x^{2}}-k_{s}G_{xz}A(\frac{\partial^{2}w_{0}}{\partial x}+\phi_{x})={\hat{I}}_{2}\frac{\partial^{2}\phi_{x}}{\partial t^{2}} \end{array}$$

To obtain the governing equation of buckling under compressive edge load, the inertia terms and applied transverse load q are both set to zero for the buckling analysis, and ${\hat{N}}_{x0}=-N_{xx}^{0}$.

(23) $$\begin{array}{l} k_{s}G_{xz}A(\frac{d^{2}W}{dx^{2}}+\frac{d\phi_{x}}{dx})+b{\hat{N}}_{x0}\frac{d^{2}W}{dx^{2}}=0\\ E_{xx}I_{yy}\frac{d^{2}\phi_{x}}{dx^{2}}-k_{s}G_{xz}A(\frac{dW}{dx}+\phi_{x})=0 \end{array}$$

Equation (23) can be solved for dϕxdx to obtain:

(24) $$k_{s}G_{xz}A\frac{d\phi_{x}}{dx}=-(k_{s}G_{xz}A-b{\hat{N}}_{x0})\frac{d^{2}W}{dx^{2}}$$

Integrating Equation (24) with respect to x and differentiating Equation (23) with respect to *x*, then substituting them for dϕxdx from Equation (24) obtains the result:

(25) $$E_{xx}I_{yy}(1-\frac{bN_{xx}^{0}}{k_{s}G_{xz}A})\frac{d^{4}W}{dx^{4}}+bN_{xx}^{0}\frac{d^{2}W}{dx^{2}}=0$$

The general solution of Equation (25) is:

(26) $$W(x)=c_{1}\sin\lambda x+c_{2}\cos\lambda x+c_{3}x+c_{4}$$

where

(27) $$bN_{xx}^{0}=\frac{\lambda^{2}E_{xx}I_{yy}}{\left(1+\frac{\lambda^{2}E_{xx}I_{yy}}{k_{s}G_{xz}A}\right)}$$

where the boundary conditions of the beam may be used to derive the integration constants *c*_1_, *c*_2_, *c*_3_ and *c*_4_.

## 4. Experimental Program

Two different tests were applied in this study: compressive and buckling tests. Two different types of samples with 0% (neat epoxy) and wt 0.3% CNT were examined. For each type of sample, two repetitions were investigated to give more accurate results.

### 4.1. Tensile Test

In order to obtain the tensile strength of the samples, the samples with nominal dimensions of 250 mm × 25 mm × 3 mm (length x width x thickness) were tested under tensile forces using a universal testing machine with 10-ton capacity. The distance between the gages was 200 mm. The procedure given in ASTM D3039 was utilized. The forces were applied at the speed of 1 mm/min.

### 4.2. Compressive Test

**Figure 4:**
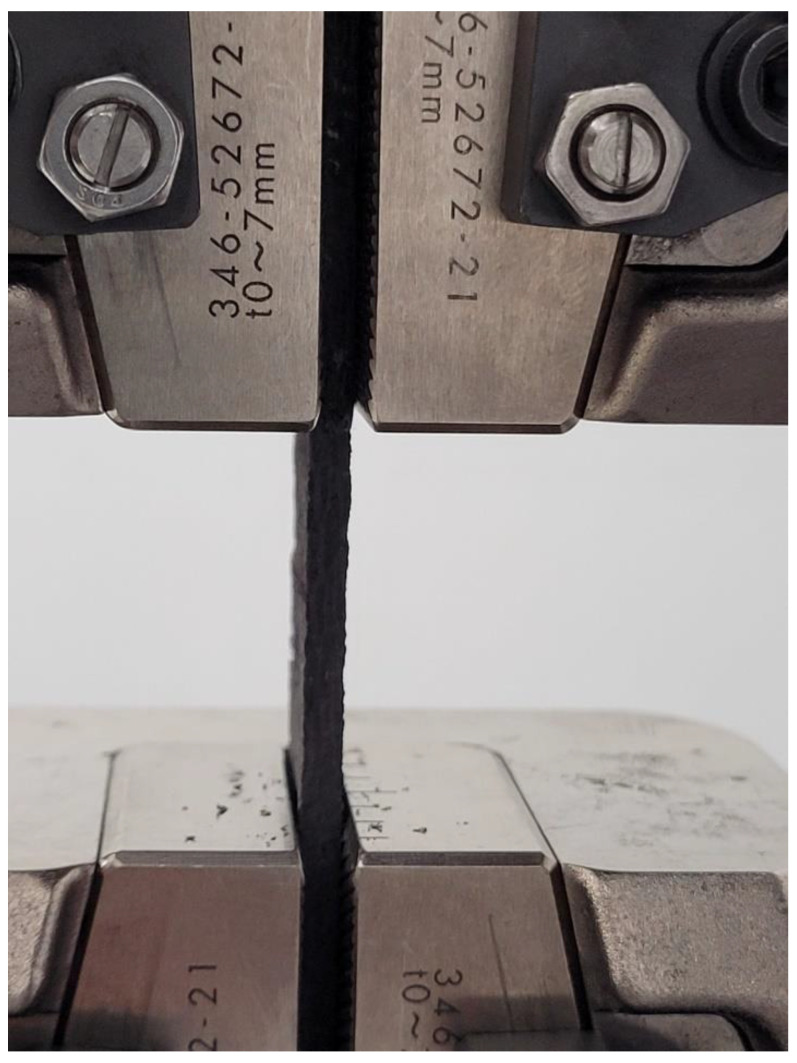
Compression test setup.

In order to obtain the compressive strength of the samples, the samples with nominal dimensions length 150 mm, width 25 mm and thickness 3 mm were exposed to axial compression forces using a Shimadzu universal testing machine with a capacity of 10 tons. The distance between the gages was 40 mm. The procedure given in ASTM D695 was applied. The forces were applied at the speed of 1 mm/min. The test setup utilized in this study is presented in Figure 4.

### 4.3. Buckling Test

**Figure 5:**
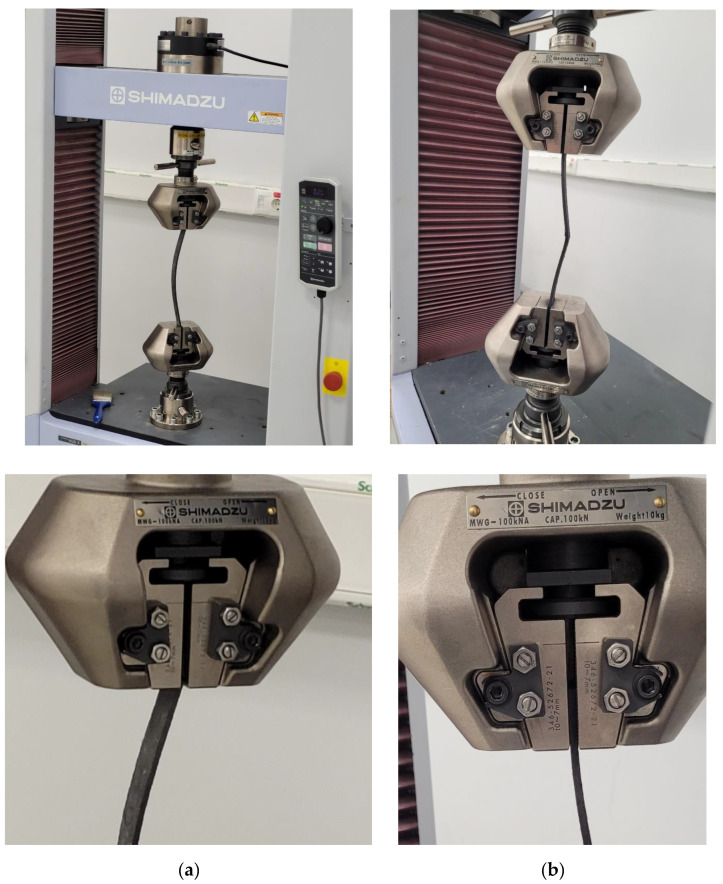
Test setups depending on boundary conditions. (**a**) simple–simple; (**b**) clamped–clamped.

The samples were examined under compression loads to determine the buckling behavior of the samples. The nominal dimensions of the specimens were 250 mm × 25 mm × 3 mm. The samples were tested with the same test machine. Similar to the compression test, buckling tests were carried out with a load speed of 1 mm/min. Utilizing clamped–clamped and simple–simple boundary conditions, the buckling performance of the samples was examined. Claw spacings of 200 mm and 250 mm were chosen for the clamped–clamped and simple–simple boundary conditions, respectively. The tests setups for different boundary conditions are shown in Figure 5.

## 5. Results

The material properties of the samples produced using micro-mechanical models were determined analytically, and the outcomes are presented in Table 2 in comparison with the experimental findings.

For the buckling analysis of the samples, the analytical solutions based on Timoshenko theory were in good agreement with the buckling results of the samples obtained by experiments, as shown in Table 3 and Table 4.

**Figure 6:**
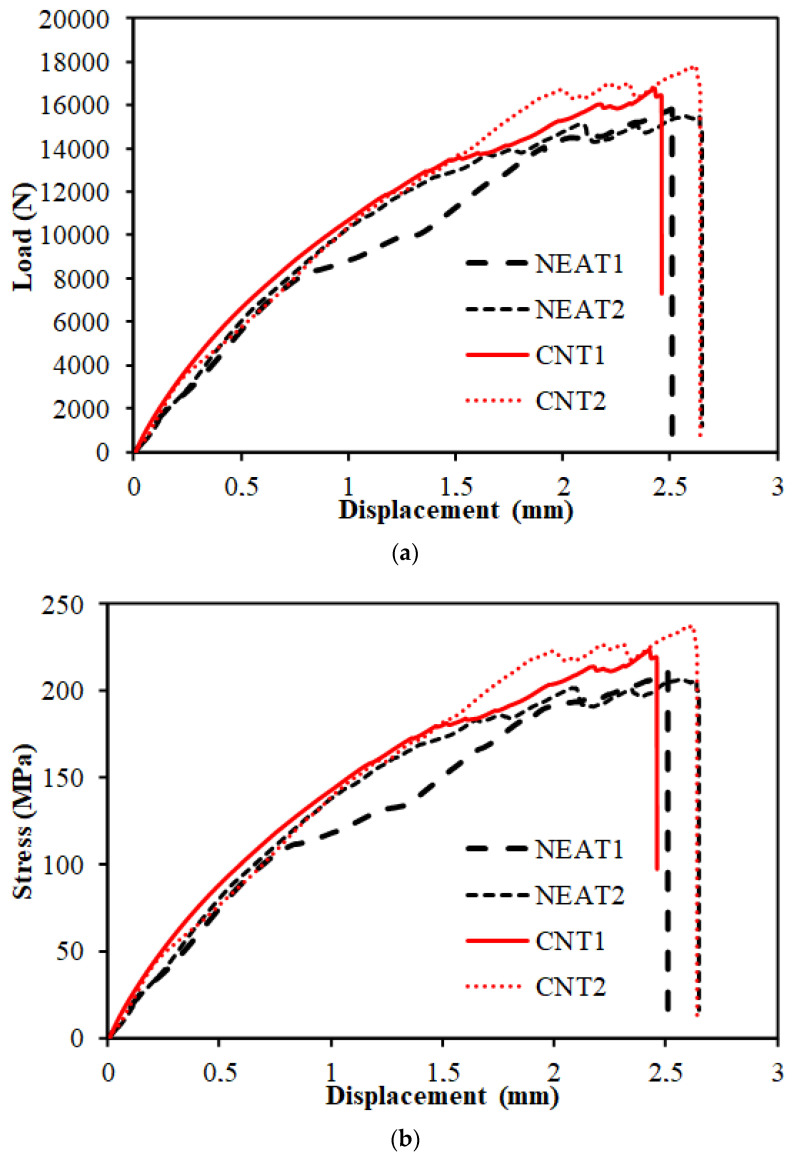
(**a**,**b**) Compression test results of the samples.

**Figure 7:**
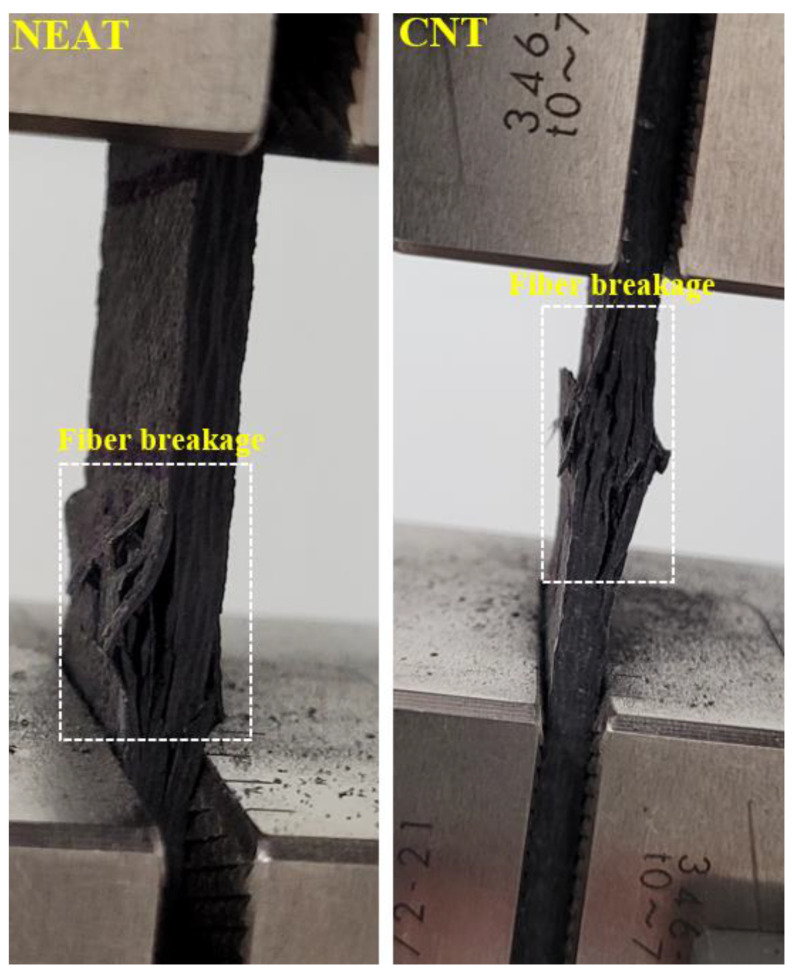
Macro damage in the samples.

Load–displacement curves of the compression tests are depicted in Figure 6. The results demonstrate that the average compressive strength of CNT1 and CNT2 at the time of collapse was 17,291.76 N, while that of NEAT1 and NEAT2 was 15,661.21 N. Maximum compressive load values of CNT1 and CNT2 were 6% and 8.10% higher than those of NEAT1 and NEAT2, respectively, while CNT2 was 12.6% and 14.80% higher (Table 5). That is, it was determined that the samples with CNT carried an average of 10.4% more load than the samples with NEAT. All specimens reached the collapse position after approximately 2.5 mm of displacement. Another issue to be considered when comparing the samples with CNT and samples with NEAT is that the displacement amounts were the same under these loads. In other words, samples with CNT carried more load for the same displacement. In addition, the ratio of the maximum load value to the cross-sectional area of the sample (25 mm × 3 mm) and the stress values were also calculated (Figure 6). Damage formations in the samples occurred as shown in Figure 7. The experiment was terminated by observing fiber breakage damage as a result of typical pressure crushing. The initial stiffness values, that is, the angle of the curve with the horizontal, were higher in the CNT.

**Figure 8:**
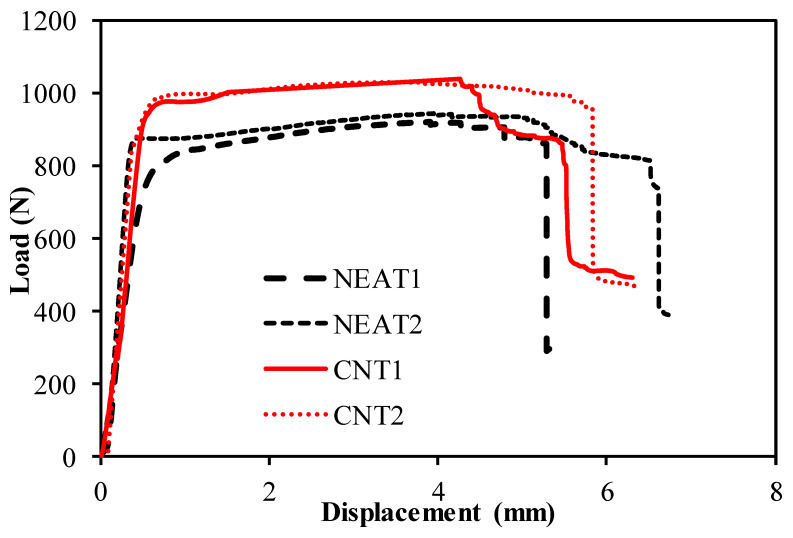
Buckling test results of the samples with simple–simple.

**Figure 9:**
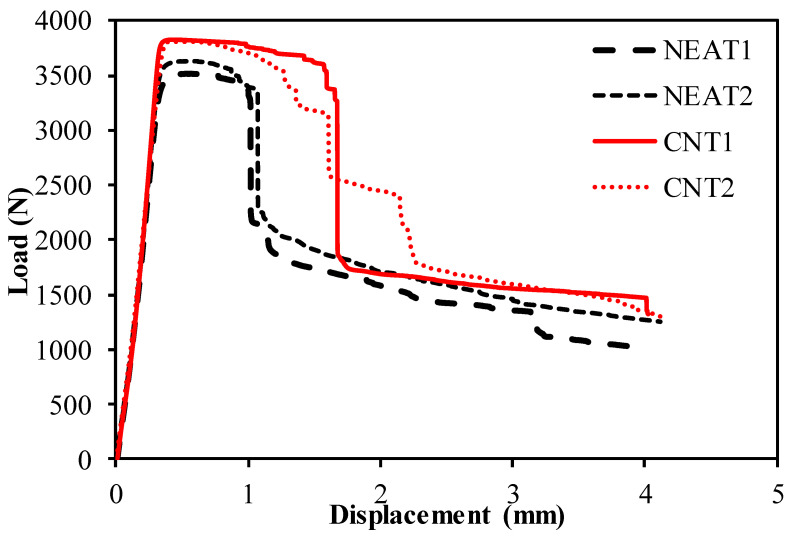
Buckling test results of the samples with clamped–clamped.

The buckling load–displacement plots of the specimens are shown in Figure 8 and Figure 9. The relationship for simple–simple is given in Figure 8, and the relationship for the clamped–clamped situation is given in Figure 9. When Figure 8 is examined, it can be seen that the mean buckling load value of CNT1 and CNT2 was 1036 N, while the mean buckling load value of NEAT1 and NEAT2 was 933 N. Maximum buckling load values of CNT1 and CNT2 were 12.8% and 10% higher than NEAT1 and NEAT2, respectively, while CNT2 was 11.8% and 9.1% higher (Table 6). In other words, samples with CNT carried a 11% greater buckling load on average than samples with NEAT. All specimens had vertical displacements between 5.48 mm and 6.73 mm due to buckling. This shows that the simple–simple supported CNT specimens achieved similar displacement with a higher buckling load. When Figure 9 is examined, it can be seen that the mean buckling load value of CNT1 and CNT2 was 3817 N as a result of the buckling test, while it was calculated as 3572 N for NEAT1 and NEAT2. The average load-carrying capacity for the clamped–clamped boundary condition was 268% higher in the CNT samples and 282% higher in the NEAT samples compared to the simple–simple condition (Table 7). Up until the critical buckling threshold, the specimens showed a more rigid behavior as a result of the clamped–clamped boundary conditions.

**Figure 10:**
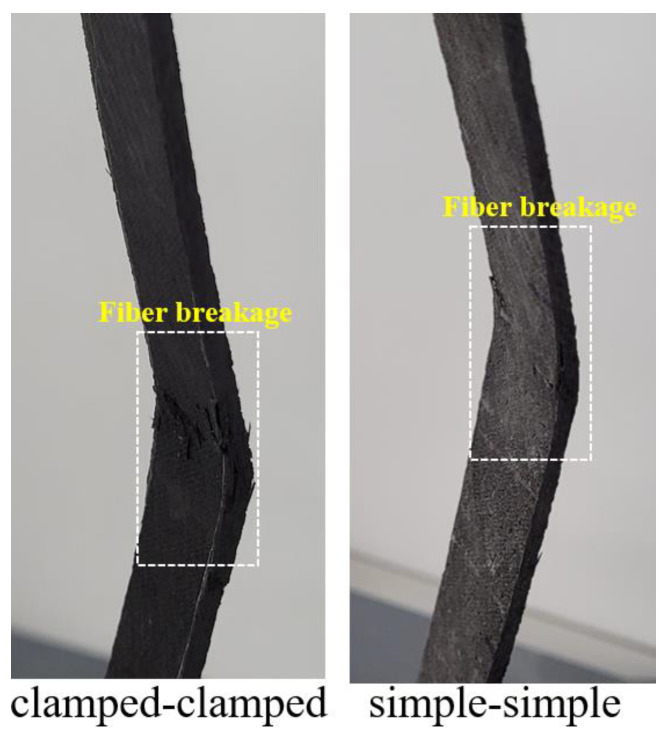
Damage analysis of simple–simple and clamped–clamped supported samples.

Final damage views on the samples are given in Figure 10. The final damage in both support boundary conditions was fiber breakage. However, this situation was observed more clearly in the clamped–clamped boundary condition.

## 6. Conclusions

In this paper, the impact of CNTs on the buckling analysis of CNTRC beams was analyzed analytically and experimentally. The mechanical behavior of composites was considerably altered by small CNT ratios. Considering the literature, significant improvements were obtained in the case of low CNT ratios.

- The experimental outcomes indicated that adding CNT can improve a composite’s mechanical performance by up to 0.3%;
- The developed analytical model and the previously presented experimental results exhibited great agreement;
- The average load-carrying capacity for the clamped–clamped boundary condition was 268% higher in the CNT samples and 282% higher in the NEAT samples compared to the simple–simple condition. Therefore, the supporting conditions of the samples caused the capacity and load–displacement curves of buckling behavior to change;
- The damage modes that occurred after compression and buckling were intense fiber breakage. In other words, in all buckling tests, it was observed that the damage initiated with micro cracks and showed progressive damage in the form of fiber rupture.

This study was limited to CNTRC beams and to the compression and elastic buckling behavior. In further research, many different types of laminates could be employed.

## Figures and Tables

**Table 1:** Material properties.

	Carbon Fiber	Epoxy Matrix	CNT
*E*_11_ (GPa)	230	2.72	500
*E*_22_ (GPa)	15	2.72	500
*E*_33_ (GPa)	15	2.72	500
ʋ_12_	0.28	0.3	0.26
ʋ_13_	0.28	0.3	0.26
ʋ_23_	0.28	0.3	0.26
*G*_12_ (GPa)	15	1.18	
*G*_13_ (GPa)	15	1.18	
*G*_23_ (GPa)	15	1.18	

**Table 2:** Young’s modulus model results.

Sample	Young’s Moduli (GPa)	
	Experimental	Analytical
0.0%wt CNT	12.18	14.48
0.3%wt CNT	13.42	16.18

**Table 3:** Comparisons of critical loads for simple–simple CNTRC beams.

	0%	0.3%
Experimental	933	1036
Analytical	947	1102

**Table 4:** Comparisons of critical loads for clamped–clamped CNTRC beams.

	0%	0.3%
Experimental	3572	3817
Analytical	3593	3899

**Table 5:** The maximum compression load and displacements of samples.

Sample	Maximum Load (N)	Rate of Increase (%)	Maximum Displacement (mm)
NEAT1	15,816.18	—	2.51
NEAT2	15,506.25	—	2.65
CNT1	16,771.16	6 and 8.10	2.46
CNT2	17,812.36	12.10 and 14.80	2.64

**Table 6:** The buckling load and vertical displacements of simple–simple supported samples.

Sample	Maximum Load (N)	Rate of Increase (%)	Maximum Displacement (mm)
NEAT1	922.20	—	5.48
NEAT2	945.28	—	6.73
CNT1	1040.44	12.8 and 10	6.30
CNT2	1031.68	11.8 and 9.1	6.35

**Table 7:** The buckling load and vertical displacements of clamped–clamped supported samples.

Sample	Maximum Load (N)	Rate of Increase (%)	Maximum Displacement (mm)
NEAT1	3516.02	—	3.88
NEAT2	3629.42	—	4.11
CNT1	3825.80	8.8 and 5.4	4.02
CNT2	3808.21	8.3 and 4.9	4.13

## Data Availability

Not applicable.
